# High-Density Lipoproteins in Non-Cardiovascular Diseases

**DOI:** 10.3390/ijms23169413

**Published:** 2022-08-20

**Authors:** Ilaria Zanotti

**Affiliations:** Dipartimento di Scienze Degli Alimenti e del Farmaco, Università di Parma, 42124 Parma, Italy; ilaria.zanotti@unipr.it; Tel.: +39-0521-905040

High-density lipoproteins (HDLs) represent physiological carriers of lipids and proteins, the activity of which has been related to cardiovascular health for decades [1]. Research efforts have focused on elucidating the underlying mechanisms that account for such benefits, revealing that several properties, namely antioxidative, anti-inflammatory, and antiaggregant effects, as well as the capacity to promote cell cholesterol efflux, are involved [2].

Recent compelling data demonstrated that alterations in size and composition, leading to the appearance of dysfunctional particles [3], may contribute to the development of a wide array of non-cardiovascular diseases.

This Special Issue entitled “High-Density Lipoproteins in Non-cardiovascular Diseases” collected original articles and reviews addressing the role of these lipoproteins in neurodegenerative disorders, cancer, pulmonary or renal diseases, and pathogen-driven pathologies.

Two papers focus on the impact of HDLs on renal diseases.

The review by Linton’s group [4] summarizes the most recent evidence supporting the reciprocal interactions between kidney functions and HDL homeostasis. The kidneys are important regulators of HDL metabolism, and these particles, in turn, profoundly affect the organ structure and function by modulating the cell cycle, differentiation, proliferation, apoptosis, inflammation, and oxidative stress. In a setting of renal failure, HDLs present altered lipidome, proteome, and small RNAs cargo and are characterized by impaired cholesterol efflux capacity [5], proinflammatory, oxidant [6], and antiangiogenic [7] activities. Observational [8] and Mendelian randomization [9] studies further support the relationship between dysfunctional HDLs and renal disease outcomes, while cholesterol efflux capacity has been demonstrated as a reliable predictor of renal transplant failure [10]. Based on this evidence, the authors finally present novel HDL-based therapeutical strategies to treat kidney disease. Although promising preliminary data have been derived from studies on patients affected by acute kidney injury [11], the validity of this approach regarding patients affected by chronic renal failure is still under investigation.

As mentioned above, it is generally accepted that HDLs turn into a pro-inflammatory phenotype in renal disease, but the characterization of their activity is only partial. In his research paper [12], Cohen aims to further contribute to this field by evaluating how HDLs collected from patients with chronic kidney disease (CKD) or hemodialysis (HD) influence the function of polymorphonuclear leukocytes (PMNLs), the primary cells of the native immune system [13]. The insights derived from this study underline the complexity of the role of HDLs in pathological conditions: HDLs from uremic patients led to an increase in CD14 expression in PMNLs, independently of the content of serum amyloid A. Surprisingly, the same effect, although to a lesser extent, was observed when cells were treated with HDLs from healthy subjects. Conversely, HDLs from uremic, but not healthy subjects, reduced the activity of Rac1, a small protein involved in the host response to pathogen infections [14]. In conclusion, although some novel characteristics of HDLs in the kidney diseases has been unraveled, more research is needed to fully understand the role of dysfunctional HDLs in the increased inflammation and risk of cardiovascular disease experienced in these diseases.

The multifaceted implications of HDLs with pulmonary functions have been addressed by two reviews: the first, by Kotlyarov, focuses on the ATP-binding cassette A1 (ABCA1)-mediated effects of HDLs in the pathogenesis of the chronic obstructive pulmonary disease (COPD) [15]. As generally accepted, this transporter plays a key role in mediating the best-characterized antiatherogenic function of HDLs, namely the initial step in the promotion of reverse cholesterol transport [16]. ABCA1 specifically expressed on alveolar macrophages [17] is implicated in the development of COPD, a heterogeneous pathology in which disorders of lipid metabolism and inflammation both contribute to the onset of clinical manifestations [18]. The author summarizes the multiple mechanisms accounting for such involvement and provides an overview of the studies through which molecular details have been unraveled. The reduction in ABCA1 expression observed in COPD patients [19] is accompanied by the loss of anti-inflammatory function [20,21], impaired RCT, and angiogenesis [22]. Taken together, these alterations may primarily contribute to the pathogenesis of COPD [23] and the development of clinically specific phenotypes [24,25]. Despite not being directly assessed, the studies reporting the role of ABCA1 in COPD provide evidence that points to the potential role of HDLs, as the physiological activators of this transporter, in the disease. The currently available data present a complex picture, in which the association of HDL levels with the disease severity is ambiguous. In fact, some studies associate high levels of HDL with compromised respiratory function [24], whereas others associate low levels with the severity of the disease [26]. Further studies are necessary to better characterize lung ABCA1 as a promising pharmacological target for pulmonary function.

The review by Trigatti et al. [27] explores the complex role of these lipoproteins in the SARS-CoV-2-mediated infection. The observation that subjects affected by COVID-19 present lower levels of HDL [28] and that an inverse correlation between particle concentrations and disease severity exists [29] drove in vitro studies, by the authors themselves as well as other colleagues, in the attempt to explore this link. Interestingly, cellular mechanisms were unveiled: On the one hand, low HDL concentrations promote virus entry into cells, thus promoting the infection. On the other hand, higher levels of these lipoproteins impair this process [30], thus protecting cells from infection. In both cases, the scavenger receptor class B type I (SR-BI) is likely to play a crucial role in mediating HDL effects. The role of this receptor in the interaction between viruses and cells is no longer in doubt [31], but the clinical implications are still under investigation. An additional aspect revealed by the authors is the alteration in HDL composition in COVID-19 patients [32], although the actual impact on particles’ function has not yet been directly addressed. In conclusion, the uncovered data are likely to identify HDLs as promising targets for their beneficial effects in addressing this global health emergency.

The pivotal role of SR-BI I in mediating HDL activities is also explored in the setting of neuroprotection by Meilhac’s group [33]. The authors report original data derived from in vitro and in vivo studies, employing endothelial cell cultures and transgenic mice. In the former model, the contribution of HDLs to the integrity of the blood–brain barrier was evident in SR-BI-expressing cells but not when the receptor was blocked by a pharmacological inhibitor. In the latter, the authors demonstrate that the expression of SR-BI in endothelial cells protected mice from the clinical manifestation of a stroke induced by the transient occlusion of the middle cerebral artery. In fact, the administration of HDLs reduced the infarct size in control mice but not in animals in which SR-BI was deleted.

The implications of HDLs in colorectal cancer (CRC) are the subject of a comprehensive review by Zeljkovic’s group [34]. The data revealed in this paper establish an inverse association between HDL cholesterol concentrations in the plasma and colon cancer development and severity/survival [35]. However, this association may be weakened depending on the specific anatomical localization of the tumor, the phenotypic characteristics, the stage, and the simultaneous adjuvant therapy [36,37]. The authors highlight the lack of consistent data characterizing the compositional alterations of HDLs in this disease, since only few, small-sized studies have been carried out thus far. Therefore, the impact of changes in HDL proteome on the functions of HDL particles and their implication in CRC disease is far from being elucidated. The available data likely point to an inverse, causal association of apoA-I with CRC development and severity [38], due to the apolipoprotein antitumorigenic, immunomodulatory activities established in vitro and in vivo [39,40]. Conversely, the role of other protein components such as apolipoprotein M [41,42], paraoxonase 1 [43,44], cholesteryl ester transfer protein (CETP) [45,46] and lecithin cholesterol acyl transferase (LCAT) [46] is more controversial and deserves further investigations. In addition, the authors present studies in which the genetic and epigenetic changes in relevant HDL regulators, such as ABC transporters, SR-BI, LCAT, and CETP, are possibly linked to CRC. Given the established role of cholesterol metabolism in tumor development, the impact of those factors affecting cholesterol availability for cell growth and replication in tumor development is not surprising. Studies assessing the expression of the above-mentioned genes/proteins in CRC tissues reveal them as potential diagnostic or prognostic markers of the disease [47,48,49], although more studies are necessary to fully support this proposal. The last section of the review deals with the potential benefits of HDL-based therapeutic procedures in CRC. Currently, promising results have been derived from preclinical studies, according to which the utilization of the modulators of ABCA1 expression [49,50], apoA-I mimetics [51], or HDL nanoparticles [52] resulted in the inhibition of tumor cell proliferation rate and a reduction in tumor growth in vivo. This very interesting topic warrants future investigations to solidify the nature of the relationship between HDL and CRC and to define new therapeutic targets for the treatment of this malignancy.

As a comprehensive summary of this collection, the review by Frikke-Schmidt’s group reports data from observational and human genetic studies exploring the relationship between HDL cholesterol and age-related macular degeneration (AMD), dementia, type II diabetes, and infections [53]. AMD represents an example of a disease in which high levels of HDL cholesterol are associated with increased risk, as shown by multiple observational studies [54]. Strikingly, Mendelian randomization studies suggest a causal relationship between these factors, possibly related to the genetic variants of *CETP* and apolipoprotein E (*APOE*) genes [55]. The mechanisms underlying this association are poorly understood, but they are possibly related to the role of HDLs as cholesterol transporters in the retinal pigment epithelium [56]. The association between HDLs and dementia is more controversial since both high and low levels of HDLs have been associated with increased risk in observational studies [57,58]. Genetic studies clearly correlated the loss-of-function mutations of ABCA1 and low levels of apoE with an increased risk of Alzheimer’s disease, possibly leading to the impaired transendothelial clearance of apoE4-beta-amyloid, with the consequent accumulation of amyloid plaques [59]. Although more studies are necessary to evaluate the impact of HDL levels on other types of dementia [60], the role of dysfunctional particles, less able to provide cholesterol to the brain in dementia pathogenesis, is no longer in doubt. The correlation between HDLs and the onset of diabetes has been the subject of several studies, clearly demonstrating an inverse association between low levels of these lipoproteins and high risk of the disease [61]. Modifications of HDL structure and function in the setting of altered glucose metabolism play pivotal roles in the onset and progression of diabetes [62], but Mendelian randomization studies do not provide evidence supporting a causal association between genetically low levels of HDL and an increased risk to develop this disease [63,64].

## Conclusions

Despite the traditional view of HDLs as carriers of “good” cholesterol, with protective activity toward cardiovascular diseases, multiple lines of evidence highlight a more complex picture, where HDLs play critical roles in several diseases, with unpredicted outcomes.

A better understanding of the mechanisms underlying the implication of HDLs in the development of non-cardiovascular diseases and further research on which components of these particles are involved may provide novel insights into the etiopathology of these types of diseases and uncover novel strategies for therapeutical approaches targeting HDLs.
